# Norm reinforcement, not conformity or environmental factors, is predicted to sustain cultural variation

**DOI:** 10.1017/ehs.2024.23

**Published:** 2024-12-03

**Authors:** Mason L. Manning, Bill Thompson, Thomas J. H. Morgan

**Affiliations:** 1 School of Human Evolution and Social Change, Arizona State University, 900 S. Cady Mall, Tempe, AZ 85287, USA; 2 Department of Psychology, University of California, Berkeley, Berkeley, CA, USA; 3 Institute of Human Origins, Arizona State University, 777 E University Drive, Tempe, AZ 85287, USA

**Keywords:** Cultural evolution, conformist transmission, norm reinforcement, tradition stability, punishment

## Abstract

The maintenance of cross-cultural variation and arbitrary traditions in human populations is a key question in cultural evolution. Conformist transmission, the tendency to follow the majority, was previously considered central to this phenomenon. However, recent theory indicates that cognitive biases can greatly reduce its ability to maintain traditions. Therefore, we expanded prior models to investigate two other ways that cultural variation can be sustained: payoff-biased transmission and norm reinforcement. Our findings predict that both payoff-biased transmission and reinforcement can enhance conformist transmission's ability to maintain traditions. However, payoff-biased transmission can only sustain cultural variation if it is functionally related to environmental factors. In contrast, norm reinforcement readily generates and maintains arbitrary cultural variation. Furthermore, reinforcement results in path-dependent cultural dynamics, meaning that historical traditions influence current practices, even though group behaviours have changed. We conclude that environmental variation probably plays a role in functional cultural traditions, but arbitrary cultural variation is more plausibly due to the reinforcement of norm compliance.

## Graphical abstract

**Figure:**
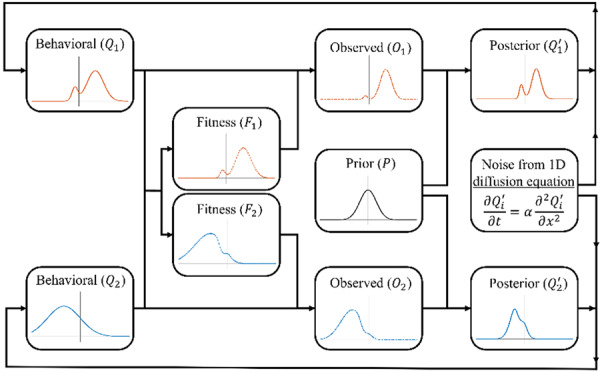

**Social media summary:** Norm reinforcement, not just payoff biases or conformity, sustains arbitrary cultural traditions.

## Introduction

Cultural evolutionary dynamics are the large-scale consequences of human interactions including how we learn from one another and how innovations arise and are spread through and between groups (Boyd & Richerson, 1985). One feature of human culture is stable between-group cultural variation, where groups might do things differently, but within any given group individuals behave similarly. Such a pattern is widespread in human societies. Thus, a key question within cultural evolution is how human traditions arise and persist, and the answer will rely on a detailed understanding of the psychological processes that guide human social learning (Boyd & Richerson, 1985; Cavalli-Sforza & Feldman, 1981).

Henrich and Boyd (1998), provide an example of cultural variation by comparing Amish farmers and more mainstream American culture. As a group, the Amish are well and widely known for their striking differences to the world around them. For instance, Old Order Amish famously do not use modern technology such as cell phones, the internet, or even electricity in their daily lives. This paired with their other cultural traditions, spanning from religion to interactions with modern medicine, creates an easily identifiable Amish culture. The distinctiveness of Amish cultural practices is all the more remarkable given their physical proximity to other populations. Another example includes the Roma, an ethnic group originating from the Indian subcontinent that can now be found around the world with significant populations residing in Europe and North America. As a group, the Roma are well known for their nomadic lifestyle and for maintaining strong cultural ties to their heritage, including distinct music, dance and language (Acton & Mundy, 1997). Furthermore, their distinctive cultural traditions persist even though they are well integrated into both urban and rural communities.

Cultural variation has also been studied in other species (Hoppitt & Laland, 2013; Rendell et al., 2011; van Leeuwen, 2021; Williams et al., 2013, 2022). For instance, van Leeuwen (2021) documented cultural variation in Chimpanzees’ grooming styles that has remained stable over at least 12 years. Nonetheless, such cases are rare and animal traditions tend to be unstable (though see Morgan & Feldman, 2024). For example, whale populations often exhibit group specific song traditions (Delarue et al., 2009; Fristrup et al., 2003; Schulze et al., 2022), but these traditions are relatively short lived, with groups innovating new songs and songs spreading between groups (Noad et al., 2000; Schulze et al., 2022; Zandberg et al., 2021). This suggests that human cultural variation is underpinned by unique psychological processes, but what these processes are remains unclear.

One mechanism widely implicated in the emergence and stability of cultural variation is conformist transmission: a disproportionate propensity to adopt cultural traits that are most frequent in the population (Boyd & Richerson, 1985; Morgan and Laland, 2012). For example, if 60% of the population has adopted some behaviour, conformist individuals are expected to adopt said behaviour with a probability greater than 60%. As a result, conformist transmission takes whichever variant is the most popular and drives it to ever higher frequencies. Theory shows that, over time, conformist transmission will homogenise groups, and where different groups converge on different behaviours, create between-group variation that can persist indefinitely (Henrich & Boyd, 1998).

The plausibility of conformist transmission underpinning cultural dynamics receives support from theoretical models which show that it is expected to evolve under a wide range of conditions, including static and spatially varying environments (Henrich & Boyd, 1998; Kendal et al., 2009; Wakano & Aoki, 2007). In addition, one distinct attribute of conformism is the ability to conform to a popular behaviour even when the payoffs associated with different behaviours are unclear. However, conformist transmission struggles in temporally varying environments (Nakahashi, 2007; Nakahashi et al., 2012) because if environmental change renders a popular behaviour deleterious, conformist transmission alone will not permit the population to adapt. Whitehead and Richerson (2009) showed that if the strength of conformity is allowed to evolve without restriction, then over time, in temporally varying environments, populations will tend towards extinction, as conformist transmission repeatedly strengthens during periods of stability, thereby preventing the spread of novel advantageous behaviours following change. Theoretical models suggest that selection favours moderate conformism, allowing other factors (such as personal information) to often override it in many circumstances (Henrich & Boyd, 2001; Kandler & Laland, 2013; Muthukrishna et al., 2016; Nakahashi, 2007; Whitehead & Richerson, 2009). In addition to theoretical support, conformist transmission is supported by empirical evidence in both adults (McGuigan et al., 2012; Muthukrishna et al., 2016) and children (Haun et al., 2012; Kohler et al., 2004; Morgan et al., 2015). For instance, Morgan et al. (2022) find that adult participants engage in conformist social learning even when aware of potential environmental changes, suggesting irrationality in the tendency. Additionally, studies on children show a developmental shift towards conformism; for instance, Morgan et al. (2015) found that children aged 3–4 were anti-conformist unless the consensus was unanimous, but by age 7, they displayed high levels of conformity. However, evidence is mixed; some adult studies show no conformist transmission (Eriksson & Coultas, 2009), while others find that the tendency strengthens over childhood but is not significant until early teenage years (van Leeuwen et al., 2018).

Thus, a combination of theoretical and empirical evidence has led to a general acceptance of conformist transmission as a proximate psychological cause of human cultural variation and tradition stability. However, other work undermines this hypothesis. Firstly, many animal studies have been conducted that find evidence for conformist transmission in other species, including primates, birds and fruit flies (Aplin et al., 2015; Danchin et al., 2018; Haun et al., 2012; Morgan et al., 2012). If human-like cultural variation is rare, but conformist transmission is widespread, this suggests that conformist transmission might not be the key factor. Secondly, cognitive science and the hybrid field of cognitive anthropology (Sperber & Hirschfeld, 2012) have documented many cognitive biases that favour certain behaviours or beliefs (Griffiths et al., 2008; Kirby et al., 2014; Reali & Griffiths, 2009). The effect of these biases becomes magnified over repeated bouts of transmission, and they can generate stable group traditions (Buskell, 2017). Morgan and Thompson (2020) added such biases to a cultural evolutionary model of conformist transmission and found that biases tend to overrule conformist transmission. They concluded that conformist transmission can only sustain between-group variation if either (1) there are no biases, or (2) conformist transmission is strong, which evidence suggests may be unlikely (Henrich & Boyd, 2001; Kandler & Laland, 2013; Nakahashi, 2007; Whitehead & Richerson, 2009). Other work has since found similar results (Yan et al., 2023).

Thus, the role of conformist transmission in generating and sustaining the between-group cultural variation seen in humans remains unclear. As such it is likely that human cultural variation is sustained by other processes, plausibly working in conjunction with conformist transmission. To this end, other mechanisms have been proposed (Henrich & Boyd, 1998), including (1) environmental variation combined with payoff-biased transmission and (2) the punishment of individuals who exhibit deviant behaviour, which we will henceforth refer to more generally as ‘norm reinforcement’.

Environmental factors drive many human behaviours (Mathew & Perreault, 2015, 2016; Towner et al., 2016). For example, the Netsilik Inuit, reflecting the climate and resource availability in the Canadian arctic coastal region, wear/wore processed animal pelts to survive the harsh winters (Balikci, 1989). Cultural evolutionary theory suggests that payoff-biased transmission, the selective copying of successful traits and individuals, will therefore lead to the spread of locally adaptive behaviours. Environmental differences between groups can thus be the source of stable between-group behavioural differences. Like conformist transmission, payoff-biased transmission receives support both from evolutionary theory (Boyd & Richerson, 1985; Kendal et al., 2009; Schlag, 1998) and human experimentation (Kendal et al., 2018; Mesoudi, 2008, 2011; Morgan et al., 2012; Vale et al., 2017). Moreover, while there is evidence of payoff biased transmission in other species (Vale et al., 2017), it is not as well documented as is conformist transmission and so there remains the possibility that differences in how payoff biased transmission occurs could be responsible for the unique scale and durability of human traditions.

Group norms that regulate human behaviour, along with reinforcement of these norms, are a universal feature of human groups (Hill, 2009). For example, Turkana men are expected to engage in dangerous cattle raids against other groups (Zefferman & Mathew, 2021), and those who fail to do so are beaten by their age mates (Mathew & Boyd, 2011). Punishments such as these may be a common form of norm reinforcement. For instance, Molho et al. (2020) examined longitudinal data from a Dutch population and identified two pathways for punishment: direct (e.g. confrontation) and indirect (e.g. gossip and social exclusion). However, punishment is not the only way that norms can be reinforced. A more positive option is the selective rewarding of those who follow norms. Alternatively, where norms regulate interactions between individuals, those who follow them may interact more successfully than those who do not and so enjoy greater payoffs even if not directly rewarded (McElreath, 2018). For instance, individuals who do not share the same language will find it significantly more difficult to cooperate on many tasks. Additionally, in many cases it is arbitrary whether norm reinforcement mechanisms are categorised as punishment or reward. For instance, among the Ache, hunting is a major source of food and is regulated by many norms. After a successful hunt, meat is shared among all adult group members. Teenage boys who join the hunt will also receive an adult share, but those who abstain may not receive anything (Hill, 2002). Whether one describes this as the rewarding of boys who hunt or the punishment of those who do not is arbitrary. The important factor is that payoffs are different for those who follow norms and those who do not, hence our use of the more general term ‘norm reinforcement‘.

Once norm reinforcement occurs, payoff-biased transmission will then favour copying from individuals who follow norms, and thus norms can be stabilised, including those that are costly to individuals (Henrich & Boyd, 2001). Because the payoff gradient is generated by the group members themselves, it does not depend on local environmental conditions and so could in theory apply to any cultural trait. Moreover, reinforcement of group norms is virtually absent in all other species (Hill, 2009; Riedl et al., 2012). Thus, norm reinforcement has the potential to contribute to the emergence and stability of human cultural variation.

In this work, we examine the ability of (1) payoff-biased transmission and (2) norm reinforcement to sustain traditions and cultural variation in the face of shared biases that would erode them. In the first case, it is sufficient to analyse a single group in isolation, since in this model we do not consider interactions between groups affecting fitness. However, in the latter case, we consider a population composed of multiple groups as norm reinforcement depends on both within- and between-group interactions. In addition, given the extensive documentation of conformist transmission, we evaluate these proposed mechanisms alongside conformist transmission, and not as an exclusive alternative. That is, we ask whether these mechanisms, in concert with conformist transmission, can maintain cultural variation, even in the face of evolved biases. Finally, below, we specifically consider continuous decisions, i.e. the choice of some value along a dimension, such as how much to contribute to a public good. For an analysis of a discrete, binary decision (which produces qualitatively similar results), see the supplementary material.

## Payoff-biased copying and environmental variation

Consider an infinitely large group of Bayesian conformist individuals who repeatedly choose a value along a continuous dimension. Let the distribution of the value in the population be normal (with mean *u* and variance *v*). While done for simplicity, this assumption of normality is reasonable because many behavioural processes are influenced by numerous small independent effects, which tend to approximate a normal distribution per the Central Limit Theorem (McElreath, 2018). Furthermore, this assumption is unlikely to meaningfully change results as the focus of the model is primarily on the mean behaviour rather than the full distribution of behaviours.

**Figure 1.**
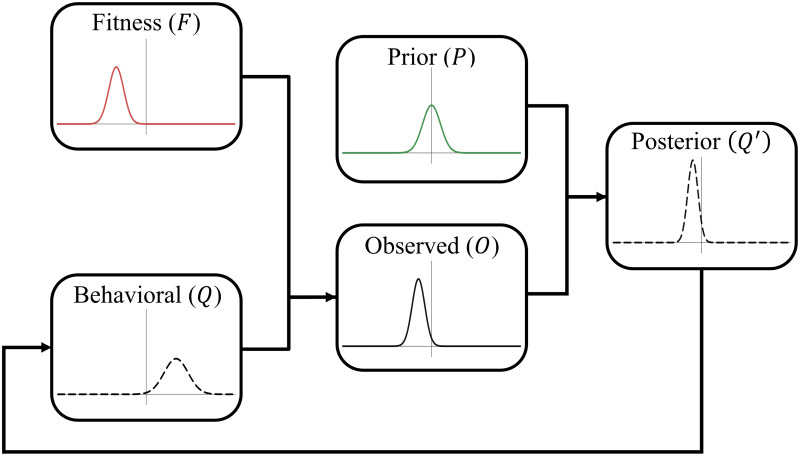
Schematic representation of the payoff-biased copying and environmental variation model. Note that group behavioural distributions are updated based on the mean of the posterior with an assumed variance of 1. See the main text for the exact formulation of each step.

Being conformists, individuals would like to adopt the mean value in the population (*u*) with an expected squared error equal to the population variance (*v*) divided by *c*, where *c* is the strength of the conformist tendency (*c*  ≥  1). Following Morgan and Thompson (2020), we assume that individuals estimate *u* by making observations of the population, which they combine with their prior beliefs (an evolved bias) according to Bayes’ rule to reach a conclusion. Note that this implementation differs from that of Boyd and Richerson (1985) where conformist individuals take *N* samples from their population's behavioural distribution and discard those outside the *x*% central confidence interval, effectively excluding ‘outliers’ from their decision-making process. In particular, this implementation can affect the mean if the distribution of values in the population is not symmetrical around the mean whereas our implementation cannot. However, given that we assume that the population distribution is symmetrical, neither implementation would change the mean value, only the variation, which will decrease. As a result, in both implementations, the variation steadily decreases, causing the population to conform to the mean value over time. For a schematic representation of this model see Figure 1.

To incorporate payoff biased transmission, each value along the dimension has a corresponding fitness that affects the likelihood an individual with that belief is observed by others. The fitness landscape is a normal distribution with mean *u*_*f*_ and variance *v*_*f*_. The distribution of values apparent to an observer (the *observed* distribution) is the true distribution multiplied by the fitness distribution, and is thus another normal distribution with mean *u*_*o*_ and variance *v*_*o*_, where:1
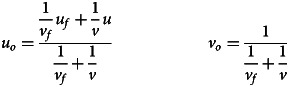


Note that the mean of the observed distribution is simply a weighted average of the means of the true distribution and fitness landscape (for the derivation of these equations, see supplementary materials).

All individuals are assumed to share the same prior belief concerning *u*, which is described as a normal distribution with mean *u*_*p*_ and variance *v*_*p*_. Conceptually, the prior is a pre-evolved bias that individuals have towards particular behaviours, created by prior natural selection. Critically, this bias is shared among all individuals, regardless of group affiliations. Generally, we expect the prior to be quite weak or uninformative on the grounds that human behaviour is highly flexible and so it is unlikely that humans have powerful biologically evolved biases for the variety of specific tasks they engage in. Rather, biases are more likely to correspond to certain abstract preferences or predispositions that have a modest impact on a wide range of behaviours. For instance, a study by Ilie et al. (2008) found that the red team in first-person shooter games was more likely to win than the blue team, despite teams being assigned through random draws of individuals. This suggests that individuals may have an evolved bias pertaining to colours, with red fostering competitive actions across contexts (Ilie et al., 2008).

Individuals then observe *N* models, sampled from the observed distribution, to update their beliefs about *u* and produce a posterior distribution. Given that the expected mean and variance of the values observed are *u*_*o*_ and *v*_*o*_, the expected posterior is a normal distribution with mean *u*′ and variance *v*′ where:2
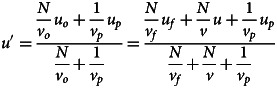
3
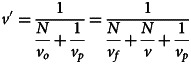


Akin to equation (1), the mean of the posterior is simply a weighted average of the means of the observed distribution and the prior, which is equivalent to a weighted average of the means of the true distribution, fitness landscape and prior (for the derivation of these equations, see the supplementary materials).

Once the posterior is calculated, individuals take the most likely value (i.e. *u*′) as their point estimate of *u* and adopt it. As per our definition of conformist transmission, individuals adopt *u*′ with an expected squared error equal to *v*ʹ/*c*, where *c* is the strength of conformist transmission. As such, after all individuals have conformed, the new value of *u* is *u*′ while *v* becomes *v*ʹ/*c*. As this process repeats, variation decreases indefinitely, while the mean value in the population will evolve to an equilibrium which we can find by identifying conditions where there is no expected change in the mean value of the population (i.e. *u* = *u*′):4
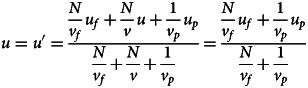


**Figure 2.**
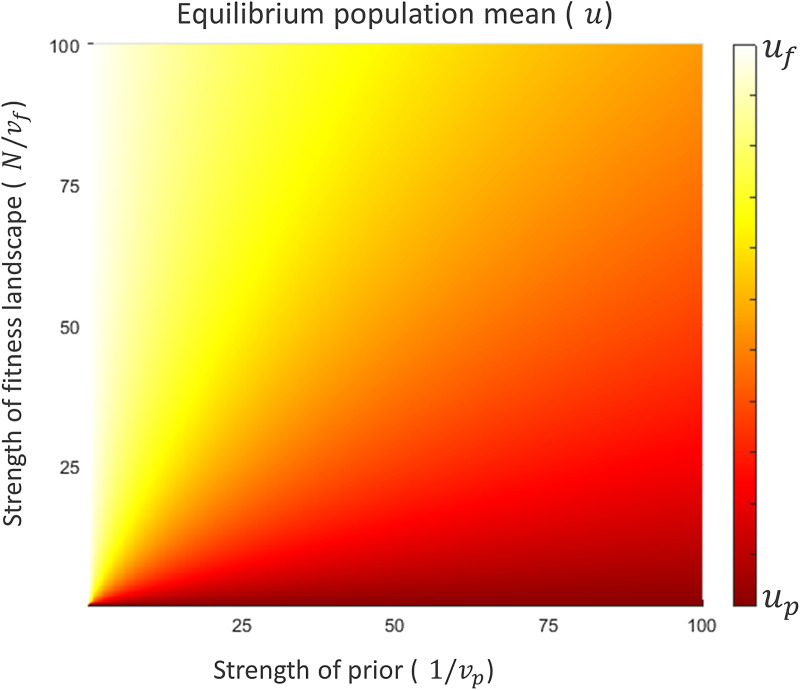
The figure illustrates the equilibrium mean value or behaviour (*u*) reached based on the strength of the fitness landscape (*N*/*v*_*f*_) and the influence of the prior (1/*v*_*p*_), where *N* is the number of observations, *v*_*f*_ is the variance in fitness and *v*_*p*_ represents the variance in the prior. The equilibrium value (*u*) consistently lies between *u*_*p*_ and *u*_*f*_. The colour coding in the figure indicates the closeness of the equilibrium value (*u*) to *u*_*p*_ and *u*_*f*_, with *u*_*p*_ shown in dark red and *u*_*f*_ in white. When the fitness landscape is weak, i.e. *n*/*v*_f_ is low, its ability to influence decision-making is limited, leading to equilibria that are largely determined by the prior, thus moving *u* closer to *u*_*p*_. This situation is akin to a world with only conformist transmission and biased priors, and as outlined by Morgan and Thompson (2020), priors dominate in this situation. On the other hand, when the prior is very weak, i.e. *v*_*p*_ is high, the fitness landscape significantly affects the equilibrium value in the population, resulting in *u* approximating *u*_*f*_.

The equilibrium mean value (4) can be interpreted as a struggle between the fitness landscape (which pulls towards *u*_*f*_ with strength *N*/*v*_*f*_) and the prior (which pulls towards *u*_*p*_ with strength 1/*v*_*p*_). Intuitively, the ability of fitness-biased transmission to drive cultural dynamics relies on both the sharpness of the fitness function and the amount of data collected. Notice that, as *v*_*p*_ approaches 0, the prior becomes infinitely powerful, resulting in the population converging on the mean of their priors. Similarly, as *N*/*v_f_* approaches infinity, the fitness landscape becomes infinitely powerful, causing the population to converge on the mean of the fitness landscape. As a result, the precise outcome depends on *v*_*f*_, *v*_*p*_ and *N*, with the equilibrium ranging anywhere between *u*_*p*_ and *u*_*f*_; as visualised in equation (5) and Figure 2 (for justification of equations (4) and (5), see supplementary materials):5
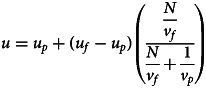

In sum, the payoff-biased transmission of continuous traditions creates equilibria that lie between values favoured by the prior (which favours *u*_*p*_ and is weighted by 1/*v_p_*) and fitness landscape (which favours *u*_*f*_ and is weighted by *N*/*v_f_*). This is a marked difference from the effect of conformist transmission alone, in which case the equilibrium is fully determined by the prior (Morgan & Thompson, 2020). However, since the equilibrium is determined by the prior and fitness landscape it can be fully explained by ecological and cognitive factors, with culture being relegated to a mediator. This mediation is evident in the updated behavioural mean value (*u*′, see equation 2) which although initially influenced by the current mean value (*u*), ultimately converges on an equilibrium dictated solely by the priors and fitness landscape (see equation 5). Therefore, cultural evolution is simply how a population evolves to reach equilibrium; it does not affect the equilibrium value itself.

## Norm reinforcement

**Figure 3.**
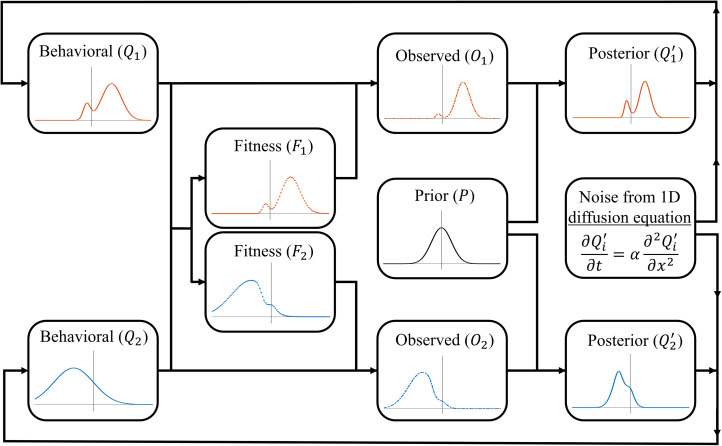
Schematic representation of the norm reinforcement model. See the main text for the exact formulation of each step.

**Figure 4.**
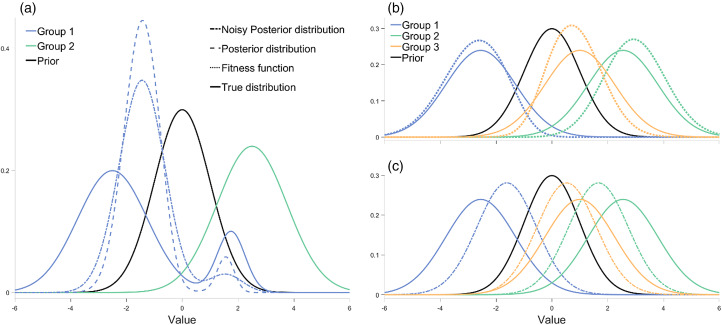
Visualisations of the model's updating process. All panels show the prior in black. (a) The behavioural distribution (solid line), posterior distribution (dashed line) and noisy posterior distribution (dot–dash–dotted line) for one of two groups (blue and green) across one timestep (the fitness function is excluded to reduce clutter). It is noteworthy that the blue population begins with a bimodal distribution, which, over time, will diminish, resulting in a convergence towards a normal distribution. However, in this particular timestep, both the posterior and the smoothed posterior retain this bimodal characteristic, which the group will adopt as their behavioural distribution in the next timestep. (b) The behaviour distributions (solid lines) and fitness functions (dotted lines) for three groups (blue, green and yellow). With more than two groups, some groups end up ‘sandwiched’ between others. (c) The behaviour distributions (solid lines) and noisy posterior distributions (dot–dash–dotted lines) for three groups. Here the prior is strong enough to pull all groups towards each other, for this timestep at least.

The specific form of norm reinforcement that we consider here is the reinforcement of social markers that indicate group membership. Such indicators, including styles of dress and linguistic markers, are widespread in human groups and are argued to have led to the evolution of an ‘ethnic psychology’ (Moya & Henrich, 2016; Moya & Boyd, 2016). In some cases, such markers may serve no purpose and be reinforced solely through deliberate punishment of those who lack them. However, in many cases such markers, even if ecologically irrelevant, can serve important *social* functions and so be highly fitness relevant even in the absence of direct punishment. For instance, where groups are hostile to one another, clearly signalling your membership can help avoid accidental hostility from your own group mates and so increase your fitness. Such contexts may be common as recent evidence supports the cultural evolutionary prediction that warfare is disproportionately directed towards culturally dissimilar groups (Handley & Mathew, 2020). Even where groups coexist peacefully, they may have different norms for regulating common problems (e.g. how food is shared after collaborative foraging). In such cases it is important to interact selectively with group mates to avoid costly disagreements and so those who reliably indicate their group membership will experience greater fitness (McElreath et al., 2003). Thus, there are multiple mechanisms, both passive and active, by which norms concerning markers of group membership can be reinforced. Refer to Figures 3 and 4 for a visual representations of the model.

Consider the same case described above with the following modifications: (1) rather than a single population, we consider *n* separate (infinitely large) sub-populations, with the behavioural distribution of the *i*th sub-population be denoted as *Q*_*i*_, with mean *u*_*i*_ and variance *v* (for simplicity, we assume that all sub-populations have the same variance, although we do not assume the distributions are normal); and (2) fitness results from the reinforcement of norms, with an individual's fitness reflecting the degree to which their value (a) correctly indicates their group affiliation
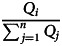
and (b) resembles behaviours typical of their group (*Q*_*i*_), the latter producing conformist effects. The fitness landscape of the *i*th sub-population, *f*_*i*_, is therefore:6
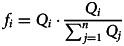


This creates different, dynamic, fitness landscapes for each group that coevolve with their compositions. Note that the most prevalent behaviours in any given sub-population do not necessarily have the highest fitness. Rather, favoured behaviours are those that are group typical, but also predictive of group membership.

Individuals use their own group behavioural distribution, weighted by fitness, in order to engage in norm-reinforcing, conformist transmission. Thus, the observed distribution, for the *i*th group, *O*_*i*_, is proportional to the true distribution in that group, multiplied by that group's fitness function:7
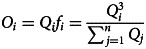
The observed distribution is not necessarily normal and has no standard form, thus it is computationally modelled, with details provided in the supplementary material.

Individuals then use Bayes’ rule to integrate the observed distribution (*O*_*i*_) and their prior. Unlike the payoff-bias model, this integration cannot be done analytically since *O*_*i*_ is a non-standard distribution. Therefore, we define *P* as the prior normal distribution with mean *u*_*p*_ and variance *v*_*p*_, and computationally calculate the expected posterior for the *i*th group using Bayes rule as follows:8
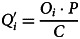
The parameter *C* in the equation represents the integration constant that normalises the product of *O*_*i*_ and *P*, ensuring that it forms a proper distribution.

Finally, noise is added to the posterior and it becomes the behavioural distribution for the next iteration of the simulation. While it might seem reasonable for groups to adopt 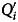 exactly as their new behavioural distribution, doing so would eventually lead to the unrealistic result of groups without any internal variation. Thus, to ensure groups have a constant variance *v*, we apply the 1D diffusion equation to smooth/flatten the posterior (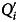), adding noise until the distribution achieves the same level of variation as the initial distribution (*v*). We denote this noisy posterior distribution as 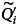. There are many ways to simulate noise; however, the 1D diffusion equation was selected because it can preserve key characteristics of the posterior, such as the mean, mode or median (see the supplementary material for more details on the heat/diffusion equation). For instance, see Figure 4a, where it adds variance to the posterior while still preserving its multi-modal features. It should be noted that this work does not explicitly investigate the mechanisms that generate within-group variation; instead, we recognise its presence and assume that our groups maintain a level of variation over time.

**Figure 5.**
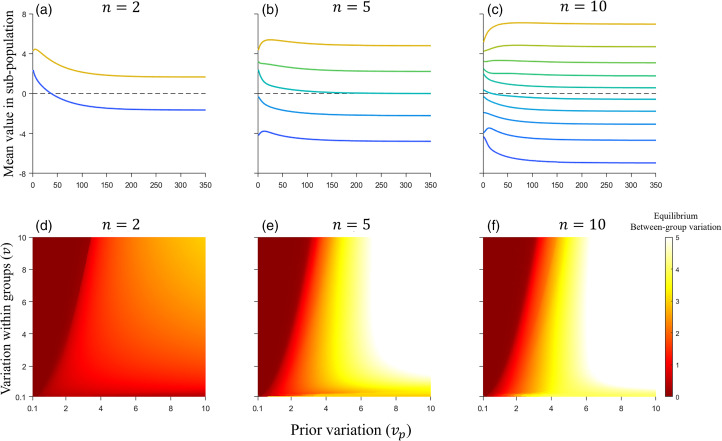
(a) Cultural evolution of two groups, assuming *u*_*p*_ = 0, *v*_*p*_ = 5 and *v* = 1. Note that at equilibrium the two groups are symmetrically distributed around a mean value of 0 which is the most likely prior value (*u*_*p*_ = 0). Note also that the group represented by the blue line crosses the peak of the prior in order to ‘get away’ from the other group. (b) Cultural evolution of five groups, assuming *u*_*p*_ = 0, *v*_*p*_ = 5 and *v* = 1. The equilibrium distribution remains symmetrical. Note that the yellow group initially moves away from more plausible prior values in order to distance itself from the green group. (c) Cultural evolution of 10 groups, assuming *u*_*p*_ = 0, *v*_*p*_ = 5 and *v* = 1. At equilibrium, groups near the peak are more tightly clustered than those on the extremities. This is for two reasons: (1) groups near the peak of the prior are surrounded by other groups that causes a cultural ‘compression’; and (2) for groups on the extremities the prior is increasingly flat and so it exerts less pressure on their equilibrium. (d–f) The equilibrium variation between the mean values of each group (i.e. between-group variation), is indicated by the colour gradient from red to yellow, with yellow signifying high variation and red signifying low or none. This variation is a function of the prior variation (*v*_*p*_) and of the variation within each group (*v*), assuming *n* =  2, 5 and 10, respectively. For each heatmap, the term ‘variation’ denotes the standard deviation between groups rather than the variance. Additionally, in (d), the equilibrium between-group variation is multiplied by 1.5 to enhance the gradient's visibility, making it easier to identify. This visual representation of the system's equilibrium states demonstrates that higher prior variation (*v*_*p*_) tends to support the preservation of between-group variation, whereas lower values facilitate the convergence of groups.

We can iterate the above equations to numerically find the equilibria of an arbitrary number of groups (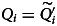). The outcome of these iterations obeys the following observations: (1) all groups are pulled towards their shared prior (Figure 5a–c); (2) the intensity of this is inversely proportional to the variance of the prior (Figure 5d–f); (3) all groups are pushed away from each other due to the fitness effects of reinforcement (Figure 5a, b); (4) the intensity of this repulsion is proportional to the number of groups and the reciprocal of within-group variance (Figure 5c–e); (5) at equilibrium the distribution of groups around the peak of the prior (*u*_*p*_) is symmetrical, with the centre of the distribution more densely populated than the tails (Figure 5a, b); and (6) regardless of the nature/shape of groups’ initial behavioural distributions, over time, they will converge towards normality (see supplementary materials).

In addition to these observations, we also explore how within-group variation and the variation in the prior affect the between-group variation. As observed in Figure 5d–f, a bifurcation exists in which groups continue to exhibit between-group variation indefinitely or collapse such that there is no between-group variation. The latter occurs when the prior's pull on groups is stronger than the repulsion from other groups, thus causing all groups to converge on their shared prior. The bifurcation is influenced by the parameters of prior variation (*v*_*p*_) and within-group variation (*v*), as well as the number of groups (*n*). As *n* increases from 2 to 10, the region sustaining between-group variation expands, suggesting that larger numbers of groups promote the maintenance of this variation. The relationship between *v* and *v*_*p*_ is non-linear, indicating that changes in within-group variation can have disproportionate effects on the equilibrium state of between-group variation.

In sum, norm reinforcement sustains cultural variation in continuous traits in the face of a biased prior, provided the prior is not overwhelmingly strong. Arbitrary starting points are typically not stable; nonetheless, at equilibrium between group differences are highly stable and average values are unique to each group.

## Discussion

The stable maintenance of arbitrary cultural traditions and cultural variation is a key question in cultural evolution. Although conformist transmission favours majority options, prior work has shown that conformist transmission alone cannot stabilise continuous traditions and cannot reliably stabilise discrete traditions when prior beliefs bias social transmission (Morgan & Thompson, 2020; Yan et al., 2023). Therefore, this work aims to investigate the roles of other social learning mechanisms, specifically payoff-biased transmission and norm reinforcement, in the stable maintenance of arbitrary cultural traditions, alongside conformist transmission and in the face of shared biases. We find that (1) the capacity of payoff-biased transmission to sustain cultural traditions is modest as it can only sustain cultural variation where such variation is manifest in the fitness landscape making cultural variation functional, as opposed to arbitrary, and (2) norm reinforcement is able to generate and maintain cultural traditions and variation, and each group converges on its own unique tradition. With norm reinforcement, culture is a key explanatory factor in human behaviour, both because the equilibria are determined by the cultural practices of reinforcement and also because the cultural history of each group matters; where groups start relative to others determines where they end up.

By creating historical contingencies in cultural dynamics, the reinforcement of norm compliance creates a route by which historical cultural practices can influence behaviour long into the future. For instance, Mathew and Perreault (2015) found that cultural history has a greater impact on many behaviours than does environment, including but not limited to economic organisation, supernatural beliefs, warfare and political organisation. One interpretation of this is that culture, as a multigenerational process, has a resulting lag as it adapts to local conditions. Indeed, experimental simulations of human culture have documented multi-generational delays as populations adapt to changed circumstances (Morgan et al., 2022), and the archaeological record suggests that while cultural change is faster than genetic adaptation, it is nonetheless far from instantaneous (Perreault, 2012). However, this work highlights the possibility of an alternative mechanism: norm reinforcement actively sustains the legacy of historical cultural practices by preventing groups from fully converging on local optima because those behaviours are already exhibited by other groups – an emergent property of the model. Under such a mechanism, it is not that these historical effects simply need more time to disappear, but rather reinforcement can sustain them indefinitely.

Another unexpected consequence of norm reinforcement is the generation of new cultural variation. Reinforcement might be expected to inhibit variation as it rewards group typical behaviours. However, as the above work shows, when groups that are culturally similar come into contact the need to maintain clear cultural boundaries drives them apart, increasing the variation across groups. This phenomenon is analogous to character displacement in evolutionary biology, where similar species diverge in response to competition for the same resources (Brown & Wilson, 1956; Pfennig & Pfennig, 2009). A possible example of this is the change of the British accent to non-rhotic (i.e. ceasing to pronounce *r's* before a consonant or at the end of words) in the eighteenth century, as a way for the ‘prestigious’ to distinguish themselves from the rest of the population (Costa & Serra, 2022), i.e. a way for the ‘prestigious’ to display their status and thus successfully interact. However, after a few decades, this dialect became mainstream, showing that groups can not only split, but also merge back together, an outcome not anticipated by our models, but which further work could address.

The potential role of reinforcement in driving human cultural evolution is emphasised by work suggesting it is a distinctly human practice. For instance, Riedl et al. (2012) found that chimpanzees do not engage in third-party reinforcement, instead choosing to retaliate over their own personal grievances or as an act of dominance. If reinforcement is indeed responsible for human cultural variation, then its uniqueness to humans is to be expected as other species do not exhibit similar levels of stable cultural variation. This can be contrasted with conformist transmission; its widespread distribution poses problems for its ability to explain unique features of human culture. Nonetheless, the generality of this work is limited by its focus on a specific form on norm reinforcement: the reinforcement of markers of group identity. Other work has considered the reinforcement of group beneficial behaviours (Henrich & Boyd, 2001). Further work could consider cases where norms regulate behaviours that serve an ecological purpose, or no purpose at all. While such work would probably be valuable, these results can make predictions about the expected dynamics. In the case where the reinforced behaviours serve an ecological purpose, provided that norms coevolve with the behaviours in the population it is likely that the resulting equilibria would mirror those in the first model, being a compromise between the environmental fitness landscape and individuals’ prior beliefs. Norm reinforcement may reduce the rate at which the population average changes (by reducing the fitness of atypical individuals), but it is not expected to affect the eventual equilibria. Where behaviours are neither group markers nor ecologically relevant, the only factors are norm reinforcement and prior beliefs, as such we expect all populations to converge on values favoured by their prior. Nonetheless, wherever behaviours are readily observable, they could become markers of group identity and thus subject to the dynamics discussed in this paper.

Although this work takes a cultural evolutionary approach (including conformist transmission and payoff biased transmission), it has also drawn from the theory of cultural attractors, which developed the idea of cognitive biases driving cultural change by incrementally transforming beliefs and behaviours each time they are transmitted (Buskell, 2017; Falandays & Smaldino, 2022; Morgan & Thompson, 2020; Sperber & Hirschfeld, 2012). Of particular relevance to this work is that cultural attraction theory has proposed additional hypotheses to explain how cultural variation is maintained, specifically ecological factors and cognitive alignment. Moreover, these two hypotheses have considerable overlap with those we explicitly considered in this work. Ecologically formed cultural attractors refer to traditions that are shaped by the environment, rather than by innate cognitive biases. Such ecological factors provide limitations or opportunities that shape the way culture is transmitted and transformed, creating a stable ‘attractor’ point that the population tends to converge on. Cognitive alignment is a culture-specific process in which individuals in a society align their beliefs and behaviours in order to interact successfully (Falandays & Smaldino, 2022). This alignment is achieved through repeated interaction and leads to the emergence of stable cultural traditions. For example, in the field of mathematics, mathematicians align their notation and symbols to communicate effectively and avoid confusion, despite knowing that notation is arbitrary.

While there are clear similarities between ecological factors and the cultural evolutionary notion of environmental variation and payoff-biased transmission, and also between cognitive alignment and norm reinforcement, there are nonetheless important differences. For instance, cultural evolutionary work emphasises that individuals learn high-payoff behaviours by copying others who are successful, often with little to no understanding of why the behaviours are successful (Boyd et al., 2011), while cultural attraction emphasises that individuals learn directly from the environment and adaptive behaviour results from multiple bouts of individuals transforming the information they acquire. Similarly, cognitive alignment refers to the process of individuals adjusting their own behaviour in anticipation of group norms through repeated interactions, while our work focuses on variation and selection by imposing negative consequences for deviating from group norms. Given these differences, the work presented here better addresses the hypotheses proposed from within cultural evolution, and although it has some overlap with those from within cultural attractor theory, further work could address the latter more directly. Moreover, empirical work will be needed to establish the roles these two mechanisms of information transmission play in human populations.

Although this study provides valuable insights into the generation and stability of between-group variation, it is important to acknowledge its limitations. Firstly, in our model of payoff-biased copying and environmental variation, we assume normality for the behavioural, fitness and prior distributions. Future work could investigate how these assumptions affect the dynamics. In particular, multi-modal priors and fitness functions may create scenarios where multiple equilibria exist and so between-group variation can be sustained. However, where distributions are uni-modal, we expect that groups will converge on the same behavioural distribution at equilibrium, resulting in no between-group variation. In the reinforcement model, we do not specify the shape of the behavioural distribution; however, we do assume that the prior is normal and there is fixed variance within groups. We anticipate that assuming a different prior would not fundamentally impact the model's conclusions. For example, if the prior were bimodal, then groups would probably cluster according to the strength of each mode in the prior, exhibiting behaviours similar to those observed while still generating between-group variation. Future studies could consider flat or uniform priors, which may produce higher degrees of variation as groups are not pulled to specific modes.

In both models, we additionally assume that (1) variation within groups is constant and (2) individuals employ the same inferential process without any population structure or specialisation (i.e. anyone can copy anyone else, all individuals are solving the same problem and with the same fitness consequences). Introducing noise is necessary because, without it, the behaviour distribution would become infinitely tight. However, future work could further explore how variation is generated within a group, including the role of individual specialisation in this process, and integrate those mechanisms into the model, making the direct addition of noise unnecessary. Finally, our model assumes the stable presence of norm reinforcement. However, reinforcement itself has evolved, and may have coevolved with markers of group identity, although, notably, chimpanzees exhibit between-group hostility, but not markers of group identity or their reinforcement. Future research could therefore explore the evolution of inter-group relations and its impact on group markers.

In conclusion, the emergence and maintenance of cultural traditions and cross-cultural variation is an important part of understanding human behaviour and what differentiates us from other species. Previous work has shown that conformist transmission alone cannot sustain traditions and variation in the face of cognitive biases. Here we show that payoff-biased transmission has a similarly limited capacity, being reliant on external environmental factors. However, norm reinforcement is a highly effective means of generating and preserving cultural traditions and variations, often leading to cases where each group converges on its own unique tradition. Norm reinforcement also creates new mechanisms for historical effects in cultural dynamics as well as the generation of novel variation. Nonetheless, further work, in particular empirical data, will be needed to establish the role of these processes in human cultural dynamics.

## Supporting information

Manning et al. supplementary material Manning et al. supplementary material

## Data Availability

n/a.

## Research transparency and reproducibility

The full model code is available at https://osf.io/6nmzw.
