# Supplementary material for: Norm reinforcement, not conformity or environmental factors, is predicted to sustain cultural variation
Source: Evol Hum Sci. 2024 Dec 3;6:e49. doi: 10.1017/ehs.2024.23 (PMC11658947; doi:10.1017/ehs.2024.23)
Supplement: Manning et al. supplementary material [file S2513843X24000239sup001.docx]

Norm reinforcement, not conformity or environmental factors, is predicted to sustain cultural variation

~ Supplementary Material ~

[Justification for equation 1 1](#_Toc47434641)

[Justification for equation 2&3 3](#_Toc47434642)

[Justification for equation 4](#_Toc47434643) 4

[Numerical estimation of *o*_i_ and *Q*’_i_ with punishment of norm violators 7](#_Toc47434647)

**A binary decision with payoff-biased transmission**

**1D diffusion/heat equation 8**

**Example of non-normal initial behavioral distribution approximating normal at equilibrium, under norm-reinforcement 10**

**A binary decision with payoff-biased copying 12**

**A binary decision with punishment of norm deviators 19**

**Brief discussion of continuous and discrete models 27**

**Justification for tipping point as conformist transmission becomes infinitely strong 30**

**Justification for equation 15 32**

## Justification for equation 1

Consider two Normal distributions: $N(u_{1}, v_{1})$ and $N(u_{2}, v_{2})$. Their product is $N(u_{3}, v_{3})$, where:

|  | $u_{3}=\frac{u_{1}v_{2}+u_{2}v_{1}}{v_{1}+v_{2}}$ |
| --- | --- |

and

|  | $v_{3}=\frac{v_{1}v_{2}}{v_{1}+v_{2}}$ |  |
| --- | --- | --- |

See: Smith, J.O. "Product of Two Gaussian PDFs", in *Spectral Audio Signal Processing,* <https://ccrma.stanford.edu/~jos/sasp/Product_Two_Gaussian_PDFs.html>, online book,

2011 edition, accessed 7/17/2020.

In the manuscript, the two normal distributions are the distribution of values in the population, $N(u, v)$, and the fitness function, $N(u_{f}, v_{f})$. Inserting these into the above we see that:

|  | $N(u, v),N(u_{f}, v_{f})=N\left( \frac{\frac{1}{v_{f}}u_{f}+\frac{1}{v}u}{\frac{1}{v_{f}}+\frac{1}{v}},\frac{1}{\frac{1}{v_{f}}+\frac{1}{v}} \right)$ |  |
| --- | --- | --- |

The mean of this Normal distribution is denoted by $u_{3}$ with variance $v_{3}$.

## Justification for equation 2&3

For a full derivation see: <https://www.statlect.com/fundamentals-of-statistics/normal-distribution-Bayesian-estimation>

Consider a normal distribution with unknown mean, *u*, but a known variance, *v*. Our uncertainty in the mean is represented by a normal prior, $N(u_{0},v_{o})$, and a series of *n* observed values, $x$, with mean, $\bar{x}$, is collected. Given this, the posterior of the mean is:

|  | $p\left( u \vert x \right)= N\left( \frac{\frac{n\bar{x}}{v}+\frac{u_{0}}{v_{0}}}{\frac{n}{v}+\frac{1}{v_{0}}},\frac{1}{\frac{n}{v}+\frac{1}{v_{0}}} \right)$ |
| --- | --- |

Or rewritten to match the notation of the manuscript

|  | $p\left( u \vert x \right)= N\left( \frac{\frac{N}{v_{o}}u_{o}+\frac{1}{v_{p}}u_{p}}{\frac{N}{v_{o}}+\frac{1}{v_{p}}},\frac{1}{\frac{N}{v_{o}}+\frac{1}{v_{p}}} \right)$ |  |
| --- | --- | --- |

## Justification for equation 4

We can solve the following with simple rearrangements:

|  | $u=u^{'}= \frac{\frac{N}{v_{o}}u_{o}+\frac{1}{v_{p}}u_{p}}{\frac{N}{v_{o}}+\frac{1}{v_{p}}}=\frac{\frac{N}{v_{f}}u_{f}+\frac{N}{v}u+\frac{1}{v_{p}}u_{p}}{\frac{N}{v_{f}}+\frac{N}{v}+\frac{1}{v_{p}}}$ |
| --- | --- |

multiply by $\left( \frac{N}{v_{f}}+\frac{N}{v}+\frac{1}{v_{p}} \right)$

|  | $u\left( \frac{N}{v_{f}}+\frac{N}{v}+\frac{1}{v_{p}} \right)= \frac{N}{v_{f}}u_{f}+\frac{N}{v}u+\frac{1}{v_{p}}u_{p}$ |
| --- | --- |

Subtract $\frac{N}{v}u$

|  | $u\left( \frac{N}{v_{f}}+\frac{N}{v}+\frac{1}{v_{p}}-\frac{N}{v} \right)= \frac{N}{v_{f}}u_{f}+\frac{1}{v_{p}}u_{p}$ |
| --- | --- |

Divide by $\left( \frac{N}{v_{f}}+\frac{1}{v_{p}} \right)$

|  | $u=\frac{\frac{N}{v_{f}}u_{f}+\frac{1}{v_{p}}u_{p}}{\frac{N}{v_{f}}+\frac{1}{v_{p}}}$ |
| --- | --- |

Write $u_{f}=u_{p}+(u_{f}-u_{p})$

$$u=\frac{\frac{N}{v_{f}}{(u}_{p}+(u_{f}-u_{p}))+\frac{1}{v_{p}}u_{p}}{\frac{N}{v_{f}}+\frac{1}{v_{p}}}$$

Distribute $N/v_{f}$ and combine like terms in the numerator:

$$u=\frac{\frac{N}{v_{f}}u_{p}+\frac{N}{v_{f}}(u_{f}-u_{p})+\frac{1}{v_{p}}u_{p}}{\frac{N}{v_{f}}+\frac{1}{v_{p}}}=\frac{\frac{N}{v_{f}}(u_{f}-u_{p})+u_{p}\left( \frac{N}{v_{f}}+\frac{1}{v_{p}} \right)}{\left( \frac{N}{v_{f}}+\frac{1}{v_{p}} \right)}$$

Divide out $\left( \frac{N}{v_{f}}+\frac{1}{v_{p}} \right)$ from $u_{p}\left( \frac{N}{v_{f}}+\frac{1}{v_{p}} \right)$ and reorder equation.

$$=\frac{\frac{N}{v_{f}}(u_{f}-u_{p})}{\frac{N}{v_{f}}+\frac{1}{v_{p}}}+u_{p}=u_{p}+\left( u_{f}-u_{p} \right)\left( \frac{\frac{N}{v_{f}}}{\frac{N}{v_{f}}+\frac{1}{v_{p}}} \right)$$

## Numerical estimation of *o*_i_ and *Q*’_i_ with punishment of norm violators

The essence of the strategy to numerically estimate *o*_i_ is to abandon the continuous value space and replace it with a discrete series of possible values. Here we will consider the set of values ranging from -20 to 20 in steps of 0.05. The distribution of values in each group, *Q*, is thus replaced with a vector of proportions at every possible value (with vector summing to 1). To illustrate, consider a group with *u* = 1. The continuous distribution is a Normal distribution with mean and variance 1. The vector distribution can be calculated with the following R code:

x <- seq(-20, 20, 0.05)

Q1 <- dnorm(x, 1, 1)/sum(dnorm(x, 1, 1))

Once this is calculated for all groups (say, 3), the fitness function for group 1 can be calculated with the following R code:

f1 <- Q1*Q1 /( Q1 + Q2 + Q3)

Then the observed distribution can be calculated as:

O1 <- Q1*f1 / (Q1*f1)

And lastly the posterior is:

*Q1’=O1*P/C*

Where C is the integration constant.

## 1D diffusion/heat equation

To maintain within-group variation after Bayesian updating of a group's behavioral distribution, we employ the diffusion equation, a fundamental concept in physics that models the spread of particles, heat, or in this case, behavioral traits across a medium over time. The diffusion equation is mathematically expressed as $\frac{\partial u}{\partial t} = \alpha\frac{\partial^{2}u}{\partial x^{2}}$, where $u(x,t)$ represents the distribution of behaviors across space $x$ and time $t$ (where $u\left( x,0 \right)=Q_{i}'$), $\alpha$ denotes the diffusivity constant indicating the rate at which behaviors spread within the group, $\frac{\partial u}{\partial t}$ is the time derivative of the distribution, and $\frac{\partial^{2}u}{\partial x^{2}}$ is the second spatial derivative showing how the distribution's shape changes across the space.

The numerical method for applying the diffusion equation involves discretizing both the spatial domain and the temporal domain into finite steps, which allows for the approximation of the continuous diffusion process on a grid where the distribution's values are known or can be calculated. A commonly used method for this purpose is the Forward Time Central Space (FTCS) scheme, chosen for its simplicity and stability given appropriate conditions.

The spatial domain is divided into a series of points with uniform spacing $\Delta x$, and the temporal domain is segmented similarly with spacing $\Delta t$. This setup forms a computational grid where each point $x_{i}$ for $i = 0, 1, ..., N_{x}$ represents a position in space, and each moment $t_{n}$ for $n = 0, 1, ...,$ represents a point in time. The discrete form of the diffusion equation can then be applied: $u_{i}^{n+1} = u_{i}^{n}+ \alpha\frac{\Delta t}{\left( \Delta x \right)^{2}} (u_{i+1}^{n} - 2u_{i}^{n} + u_{i-1}^{n})$, where $u_{i}^{n}$ is the value of the distribution at spatial point $i$ and time step $n$.

To conserve the total probability or weight of the distribution, Neumann boundary conditions are applied, ensuring no "flux" of the distribution out of the system's boundaries. This is done by setting the distribution's value at the boundaries equal to the value just inside the boundary, effectively preventing any change at the domain's edges over time.

The process begins with an initial distribution, which in this context is the posterior distribution $Q_{i}^{'}$, and iterates over time steps to simulate the diffusion of behavioral variation. After each time step, the variance of the distribution is recalculated. The iteration continues until the variance of the distribution meets or exceeds a specified minimum value, $v$. This requirement ensures that the distribution does not become overly concentrated, reflecting the real-world scenario where absolute conformity within a group is neither observed nor expected. Through this method, the distribution is adjusted to introduce noise, simulating the natural process of maintaining variation within the group and ensuring that the group's behavioral distribution reflects both the updated beliefs about group behaviors and the inherent variability of the group.

#
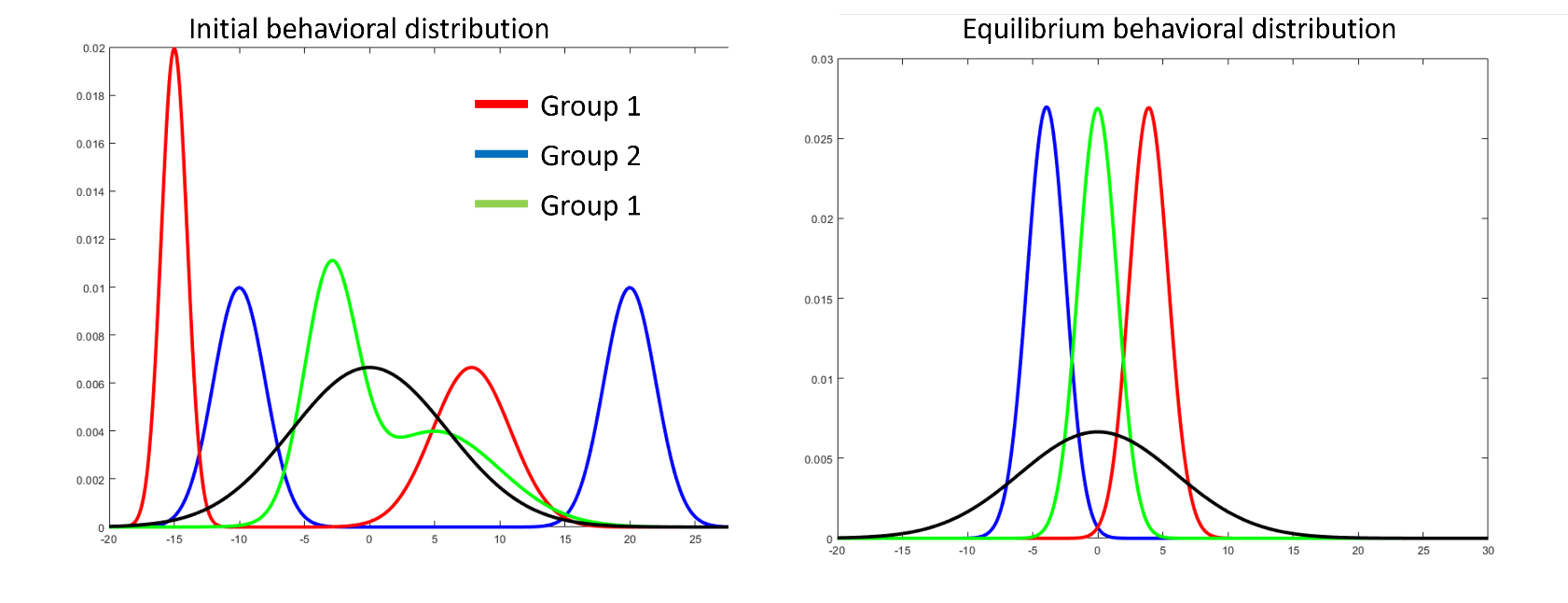
Example of non-normal initial behavioral distribution approximating normal at equilibrium. Under norm reinforcement.

Figure 0: Provide examples of three groups, each with a non-normal initial behavioral distribution. Despite this, at equilibrium, the behavioral distribution approximates normality. However, it is worth noting that, although computational findings suggest that the equilibrium distribution is always unimodal regardless of the initial distribution, we cannot prove it.

Top of Form

# A binary decision with payoff-biased copying

Consider an infinitely large group of Bayesian conformist individuals who repeatedly choose between two options; A and B. Denote $q$ as the frequency of option A in the population and subsequently $1 - q$ as the frequency of option B. Being conformists, individuals would like to conform to $q$; i.e., if $q > 0.5$ they would adopt A with probability greater than $q$. However, as in prior work (Morgan & Thompson, 2020), we relax the assumption that individuals are perfectly aware of $q$, and instead, we assume they must estimate $q$ by making observations of the population. Specifically, we assume that they make observations of the population about $q$ which can be biased in favor of a given option. We model this inference process as Bayesian learning. However, unlike prior work, we assume that observations are not made at random and that individuals engage in payoff biased transmission, i.e., the probability that an individual is observed is related to their payoff and, as such, observers may overestimate the prevalence of an option if it confers higher payoffs. In what follows we assume that A is the high payoff option and that it starts in the majority, while B is favored by biases in the inferential process. Our question is whether payoff biased transmission, in conjunction with conformity, is able to sustain such a tradition given that conformist transmission alone often cannot. To capture payoff-biased transmission, we allow the observed frequency of A ($q_{o}$) to be a function of the parameter $\tau$, which can be interpreted as the strength of payoff biased transmission; given our focus on cases where A is the high payoff option, we consider $\tau\geq1$. Alternatively, assuming transmission is directly proportional to payoffs, it could also be interpreted as the payoff of A relative to B. The relationship of $q_{o}$ to $\tau$ and $q$ is as follows:

|  | $q_{o}=\frac{q\tau}{q\tau+(1-q)}$ | (9) |
| --- | --- | --- |

Note that if payoff biased transmission is non-existent (i.e., $\tau= 1$), individuals accurately observe $q$ since, when $\tau=1$, $q_{o}=q$. Nonetheless, as the strength of payoff biased transmission ($\tau$) increases, the observed frequency overestimates the frequency of A.

As individuals are assumed to engage in Bayesian inference, they must start with a prior belief about the frequency of A. All individuals are assumed to share the same prior, which is described as a beta distribution with parameters $a$ and $b$, such that when $a$ increases higher values of $q$ are favored and when $b$ increases lower values of $q$ are favored; note when $a=b=1$ the prior is uniform. Because any bias in the prior is assumed to favor B, we consider $a=1$ and $b=1+\beta$, where $\beta$ is the bias in the prior. When there is no bias ($\beta=0$) the prior is flat i.e there is no prior preference for either option. However, as $\beta$ increases, the prior distribution is increasingly swayed towards option B (i.e., it favors low values of $q$). Individuals’ beliefs about $q$ are updated by observing $N$ other individuals (henceforth "models"), sampled according to their payoff (with $\tau$, therefore, determining the bias in sampling). The expected number of models who chose options A and B are $Nq_{o}$ and $N(1-q_{o})$ respectively. The observer’s final belief about $q$ (their posterior distribution) is therefore a beta distribution with parameters $a'$ and $b'$ where:

|  | $a^{'}=a+Nq_{o}=1+Nq_{o}$ | (10) |
| --- | --- | --- |
|  | $b^{'}=b+N\left( 1-q_{o} \right)=1+\beta+N(1-q_{o})$ | (11) |

Once the posterior is calculated, we assume individuals take the most likely value (i.e., the mode), as their point estimate of $q (q')$, giving the following:

|  | $q^{'}=\frac{a^{'}-1}{a^{'}+b^{'}-2}=\frac{N}{N+\beta}q_{o}$ | (12) |
| --- | --- | --- |

As can be seen in equation (4) the affects from $N$ and $\beta$ depend only on their relative magnitudes. Therefore, we denote $r =\frac{N}{N+\beta}$ as the proportion of the total information (data and prior) in the posterior that comes from the data, as opposed to the bias in the prior. Resulting in the following simplification of equation (4):

|  | $q^{'}=rq_{o}$ | (13) |
| --- | --- | --- |

When the number of individuals sampled is much greater than the bias in the prior (i.e., $N >> \beta$), then $r \approx1$ and the point estimate reduces to $q' \approx q_{o}$. This implies that the prior has no influence on the posterior, and instead the posterior is entirely determined by the observed frequency of A, which itself is a function of the true frequency of A ($q$) and the effect of payoff biased transmission ($\tau$). As such, when $r \approx1$ *and* $\tau= 1$ individuals accurately perceive $q$. When the bias in the prior is much greater than the number of individuals sampled (i.e., $\beta>> N$), then $r \approx0$ and the point estimate reduces to $q^{'}\approx0$. This implies that for a large value of $\beta$, the bias in the prior overwhelms the data and observers conclude that option B is popular regardless of the observations they make.

After sampling observations from the population, individuals engage in conformist transmission, adopting A with probability $p_{A}$ where:

|  | $p_{A}=\frac{q^{'c}}{q^{'c}+\left( 1-q^{'} \right)^{c}}$ | (14) |
| --- | --- | --- |

with $c$ controlling the strength of the conformist tendency. Once all individuals conform, $q$ will equal $p_{A}$ and the repetition of this process produces cultural evolution. Our goal is to identify the conditions in which an established, advantageous tradition (i.e., A with $q > 0.5$ and $\tau> 1$) can persist or even grow in the face of counteracting biases (i.e., $\beta> 1$).

Morgan and Thompson (2020) have already considered the interaction between conformist transmission and biased priors in the absence of payoff-biased transmission (i.e., $\beta> 0, c\geq1, \tau= 1$). Their results can be summarized as follows: (1) If the conformist tendency is weak, and the bias in the prior strong relative to the number of observations individuals make, the population converges on B regardless of its initial frequency. (2) If the conformist tendency is strong, the population either converges on B or reaches a mixed equilibrium favoring A. Which outcome occurs depends on the initial frequency, as well as the strength of conformist transmission and the bias. We additionally note that, for an infinitely strong conformist tendency, the tipping point between these two equilibria in terms of initial frequency is $\frac{1}{2r}$ (see supplementary material), above this value mixed equilibria are attained, below it, B reaches fixation. (3) While Morgan and Thomspson (2020) note the presence of mixed equilibria, we additionally note that once the prior is biased *to any extent* then A can *never* reach fixation and $q = 1$ is *never* stable. This can be readily understood by noting that the bias is mathematically equivalent to observing $\beta$ individuals performing option B, and so while $q'$ (and in turn $p_{A}$) can be close to 1, they can never converge on it. (4) If most of the information in the posterior comes from the bias in the prior as opposed to observations (i.e., $r < 0.5$) then the population will always converge on B, regardless of the conformist tendency or initial frequency

Let us now consider the interaction between payoff-biased transmission and the bias in the prior, but in the absence of conformist transmission (i.e., $r \leq1, c = 1, \tau\geq1$). Given that $c = 1$, the probability of adopting A ($p_{A}$) becomes equal to its perceived frequency ($q'$) and so equilibria exist where $q = q'$. This has two solutions:

|  | $q_{1}=\frac{r\tau-1}{\tau-1}, q_{2}=0$ | (15) |
| --- | --- | --- |
|  |  |  |

The first equilibrium is viable when the payoff bias is stronger than the prior bias (i.e., $\tau> 1/r$, otherwise $q_{1}$ has a negative value which is meaningless) and when this condition is met, the population will converge on $q_{1}$, otherwise B reaches fixation and $q_{2}$ is attained (see the above section, “justification for equation 15”). Note that, (1) even if $\tau> 1/r$, $q_{2}$ remains an equilibirum, just an unstable equilbirum such that any deviation from $q_{2}$ will cause the population to evolve to $q_{1}$, and (2) provided there is any bias in the prior (i.e., $r < 1$), then $q_{1}$ is a mixed equilibrium, and $q = 1$ (i.e. A fixation) is never stable.

Although $q_{1}$ illustrates that A can persist in the population, in many cases this is as a minority variant, and so the tradition is, in a sense, lost. Nonetheless, we can identify conditions for A remaining in the *majority* by finding the relationship between $\tau$ and $r$ for which $q_{1} > 0.$5. This is $\tau>\frac{1}{2r-1}$, which is simply a more stringent requirement for the strength of payoff-biased transmission relative to the bias in the prior. However, it only produces meaningful values if $r >\frac{1}{2}$and so A can only remain in the majority if observations carry more weight than the prior.

Lastly, we examine the three-way interaction between conformist tendency ($c \geq1$), relative payoff ($\tau\geq1$), and biased priors ($r \leq1$). There is no algebraic expression for the two possible non-trivial equilibria based on $c, r,$ and $\tau,$ as such we analyzed the system numerically (see Figure 1).

First let us note three outcomes that carry over from previous models: (1) When observers weight the prior sufficiently heavily relative to the information they receive (i.e., $\tau< 1/r$), B will always reach fixation, (2) Populations that start with B at fixation will always remain there, and (3) If the prior is biased *to any extent* then A can *never* reach fixation and $q = 1$ is *never* stable. Beyond this, the interaction between $c, r, \tau$ is summarized as follows: (1) The prior bias consistently draws the population towards B, but payoff-biased transmission consistently favors A. As $\tau$ or $r$ increase, the region of parameter space leading to the stable presence of A grows [see Figure 1 ($a, b, c, d$)]. (2) Intensifying conformist transmission has multiple effects: (i) for low values of $q$ and/or $r$ it can join with the bias to overwhelm payoff-biased transmission, creating cases where the mixed equilibrium is replaced by the fixed all-B equilibrium even when $\tau> 1/r$ [see Figure 1 ($b, f, j, n$)], (ii) whereas payoff-biased transmission creates a smooth gradient in the frequency of A as $r$ is varied, conformist transmission creates marked boundaries between the mixed and pure equilibria [see Figure 1 ($d, h, l, p$)], (iii) while this initially causes the region under which the mixed equilibrium is present to contract [see figure 1 ($b, f$)], further increases in $c$ can cause it to enlarge as A can persist for a wider range of values of $r$ [see Figure 1 ($f, j, n$)]. (3) For a given value of $r$, the tipping point in terms of population composition that determines whether the mixed equilibrium or all-B equilibrium is reached depends on $\tau$ and $c$; strengthening payoff-biased transmission pushes the tipping-point towards 0, favoring A, while strengthening conformist transmission pulls it away from 0, reaching 0.5 when $\tau$ and $r$ are 1, otherwise stopping at $\frac{1}{2r\tau-\tau+1}$.

In sum, payoff-biased transmission that favors a discrete tradition can enhance its stability in the face of priors that are biased against it, particularly when conformist transmission is weak. However, it cannot keep traditions at fixation and leads instead to highly mixed equilibria unless conformist transmission is strong.


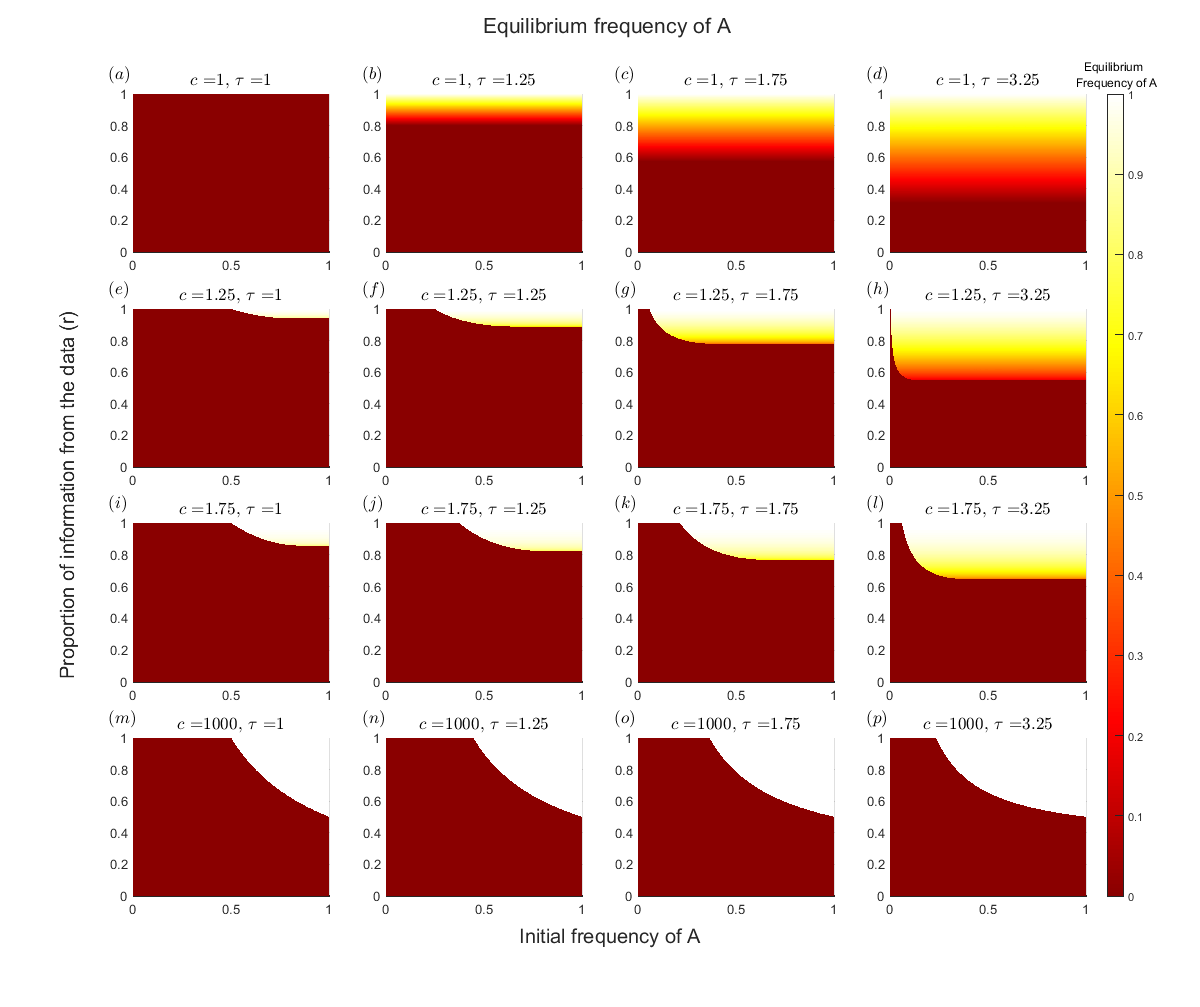
Figure 1: ($a$-$p$) Equilibrium values reached for different values of $c, r, \tau,$ and initial value ($q$). In this figure, the initial frequency of option A, and the proportion of information in the posterior that comes from the data ($r$) are the "continuous" $x$ and $y$ axis, respectively. Whereas conformist tendency ($c$) and relative payoff ($\tau$) are the "discrete" row and column subplot axis, respectively. The reached equilibrium frequency of the population is represented as color.

# A binary decision with punishment of norm deviators

We now consider the case of punishment of norm deviators, but with a binary trait. As in the previous model we consider $n$ separate (infinitely large) sub-populations, and fitness results from the punishment of norm deviators by their group mates, with an individual being punished to the extent that their chosen option does not reliably indicate which group they belong to. However, because the trait is binary, $q_{i}$ represents the frequency of option A, and $1-q_{i}$ the frequency of option B, in the $i^{th}$ sub-population. Once again, the prior is described as a beta distribution with parameters $a=1$ and $b=1+\beta$ where, $\beta,$ is the bias in the prior favoring option B.

Individuals update their behavior by sampling members of their subpopulation, weighted by fitness. Let $f_{A,i}$ be the fitness of individuals with behavior A in group $i$, $f_{B,i}$ be the fitness of individuals with behavior B in group $i$, where:

|  | | $f_{A,i}=Pr\left( A \right) =\frac{q_{i}}{\sum_{j=1}^{n} q_{j}}$ | | (16) |  |
| --- | --- | --- | --- | --- | --- |
|  | $f_{B,i}=Pr\left( B \right) =\frac{1-q_{i}}{\sum_{j=1}^{n} \left( {1-q}_{j} \right)}$ | | (17) | | |

Substituting $\tau_{i}$ as the fitness ratio $f_{A,i}/f_{B,i}$, the observed frequency of option A within the $i^{th}$ subpopulation ($q_{o,i}$) is then:

|  | $q_{o,i}=\frac{q_{i}\tau_{i}}{q_{i}\tau_{i}+\left( 1-q_{i} \right)}$ | (18) |
| --- | --- | --- |

As such, $\tau_{i}$ is effectively the punishment driven sampling bias.

Individuals update their beliefs about $q_{i}$ by observing 𝑁 other models sampled according to $q_{o,i}$. As in the previous binary model, individuals combine their observations with their priors to generate a posterior and then take the mode of this distribution as their point estimate ($q_{i}^{'}$) of the frequency of option A in their group:

$$q_{i}^{'}=rq_{o,i}$$

Where, as before, $r=\frac{N}{N+\beta}$ and can be understood as the proportion of the information in the posterior that comes from the data, as opposed to the bias in the prior. Finally, individuals engage in conformist transmission, with individuals in group $i$ adopting A with probability $p_{A,i}$ where:

$$p_{A,i}=\frac{{q_{i}^{'}}^{c}}{{q_{i}^{'}}^{c}+\left( 1-q_{i}^{'} \right)^{c}}$$

with 𝑐 controlling the strength of the conformist tendency. Once all individuals conform, $q_{i}$ will equal $p_{A,i}$ and repeating this process produces cultural evolution.

Unlike the continuous model, the results of this system depend not only on the *relative* values of $q$ in each group ($Q$ in the continuous case), but also on the specific initial values assigned to each group. This is because (1) the fitness function is contingent on the current state of all subgroups, and (2) the discrete nature of the trait along with the broadly homogenizing tendencies of conformist transmission and punishment create a fitness valley between $q_{i}=0$ and $q_{i}=1$. As such, groups can become stuck on whichever side they start, even if fitness would be higher on the other side.

To illustrate, consider a case where, at first, there is a single group, and a second group is later added. The first group does not experience fitness effects, and so evolves according to its prior and conformist transmission. For simplicity, assume the prior is weak enough that it evolves to either all-A or all-B, and that all A happens to win. The second group then appears. The prior favors all-B, conformist transmission favors homogenization in either direction, while fitness favors the opposite to the first group (so, again, B). Which option prevails depends on the strength of the prior and the amount of data collected, but also on the initial placement of the new group. If it starts sufficiently close to the first group, conformist transmission can cause it to converge on A, whereas if it starts further away it will converge on B. Similar patterns are seen as additional groups are added.

Given that the outcome depends on the starting value of each population, these $n$ initial values become $n$ additional dimensions of parameter space to consider, rendering a comprehensive exploration impossible. One way to simplify this system is to select a small number of starting conditions and then fully explore the other parameters for each set of starting conditions. Based on our interest in the ability of punishment to sustain cultural norms and cultural diversity we consider the following three cases: (1) If all subpopulations start near option A fixation (spready evenly between 0.97 and 1) can these norms be sustained? (2) If all subpopulations start near option B fixation (spread evenly between 0 and 0.03) can norms favoring option A evolve? (3) If half the subpopulations start near option A, and the other half start near option B, can this diversity be sustained?

Across all three of these starting conditions, the following universal outcomes can be observed: (1) For any given values of $c$, $n$ and $r<1$ there are at most two stable equilibria to which separate groups converge (Figures 6 [c, f, i, l]). One is B fixation and the other is a mixed equilibrium which is analytically unknown but can be estimated computationally and favors A unless the prior is very strong and conformist transmission weak (see Figures 6 [b, e, h, k]). (2) The bias consistently draws the populations towards B, both increasing the proportion of groups that converge to the all-B equilibrium and by increasing the frequency of B at the mixed equilibrium. (3) As the number of subpopulations increases so does the cultural diversity of the overall population (Figures 6 [a, d, g, j]), although this effect is small. (4) Individual groups can only converge on A fixation if $r=1$, moreover it is impossible for all groups ($n>1$) to converge on A fixation, in the absence of conformist transmission.

Considering the case where all groups start near fixation on A, if the bias is weak, the between-group similarity weakens punishment. Note the force of punishment is weak here because the initial behavioral distribution of all groups is similar, causing comparable within-group dissimilarity when conforming to B before drastically increasing between-group dissimilarity (refer to equations 12 and 13). Thus the majority of subpopulations to converge on mixed equilibrium favoring A (weak prior bias, strong conformist transmission) or a mixed equilibrium favoring B (strong prior bias, weak conformism transmission, Figure 6 [b, e, h, k]). However, in the absence of conformist transmission, at least one group will always converge on the all-B equilibrium, being driven there by punishment. Strengthening the bias, in the act of pulling populations to their prior, temporarily lessens the initially strong between-group similarity allowing punishment to further separate groups and leading to more groups converging on the all-B equilibrium (Figures 6 [a, d, h, j]). Conformist transmission sharpens the divide between the two options in two ways, (1) it greatly reduces the scope for mixed equilibria, driving groups towards all-B or moving the mixed equilibrium such that it more greatly favors A (Figure 6 [b, e, h, k]), and (2) it reduces the scope for stable cultural diversity such that groups tend to converge on all-all-B or all-mixed depending on the strength of the prior relative to the data.

When subpopulations start near option B fixation, the general patterns are the same: the bias increases the frequency of B in the mixed equilibrium and increases the proportion of groups that converge on all-B, while conformist transmission homogenizes both individual groups and the overall population. However, because groups start very close to all-B, B tends to be at higher equilibrium frequencies than in the case where groups start close to all-A. Only when conformist transmission and the bias in the prior are sufficiently weak can punishment drive a subset of groups towards the mixed equilibrium.

When half the subpopulations start near option A, and the other half starts near option B the interactions between $c, r$ and $n$ are as follows: (1) Punishment pushes the populations to stay apart. In turn keeping an even split of subpopulation between the two stable equilibria. (2) The bias in the prior can overwhelm punishment leading to majority of subpopulations conforming on option B, while also reducing the frequency of A at the stable mixed equilibria. (3) Conformist transmission has similar effects as before; homogenizing individuals’ groups and creating a sharp threshold in the strength of the prior according to which cultural diversity is either maintained or lost by all groups converging on the all-B equilibrium.

In sum, the punishment of norm deviators in the context of discrete traditions can sustain long term cultural variation, and, in conjunction with conformist transmission, sustain traditions against the effects of biased priors.


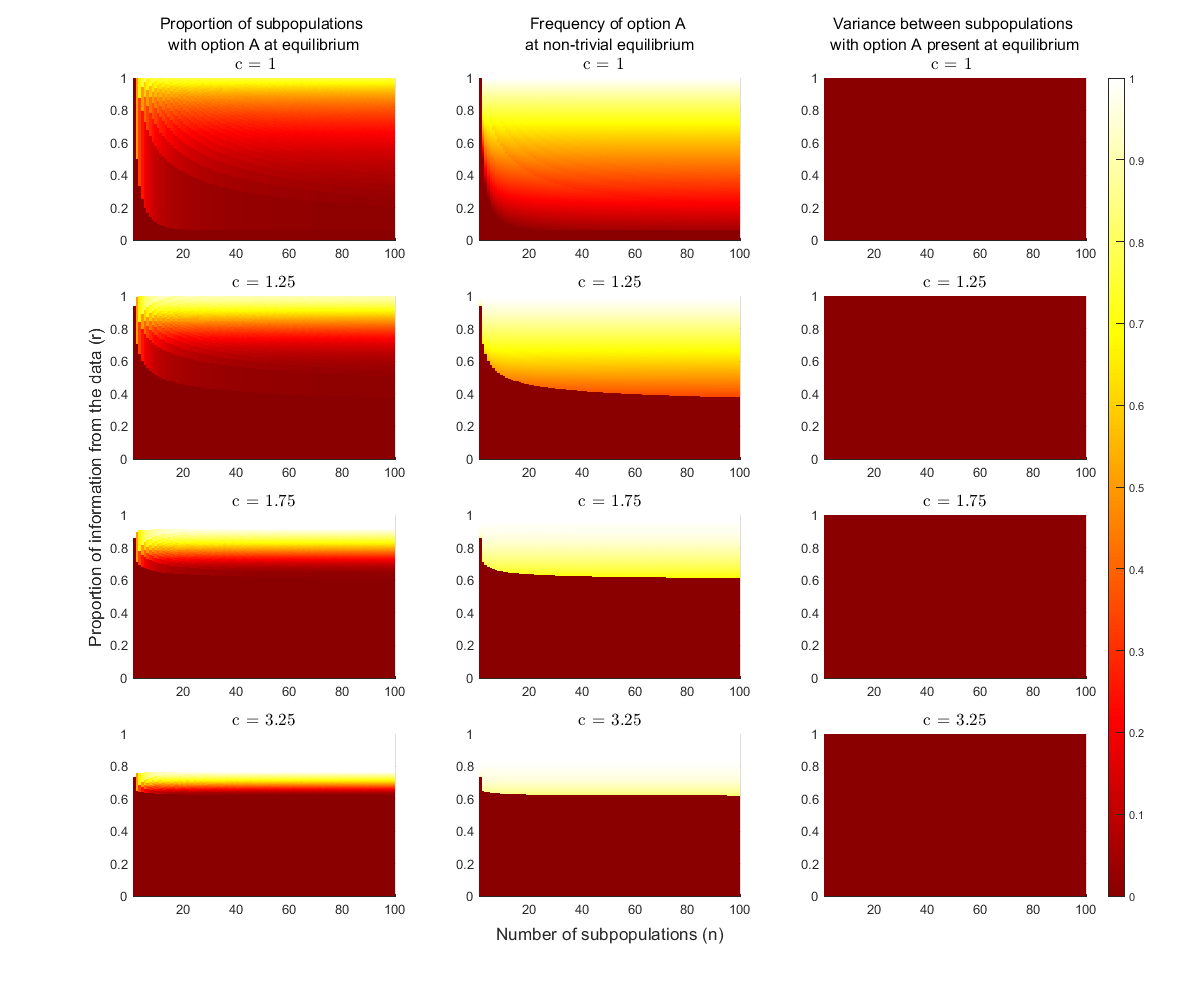


Figure 6: ($a$-$l$) Equilibrium values reached for different values of $c, r, n$. In this figure, every subpopulation is assumed to start near option A fixation, specifically $q_{i}\in(0.97,1)$ with no subpopulation starting at the exact same frequency. The first column of heatmaps uses color to represent the proportion of subpopulation that stabilized at a non-option B fixation equilibrium. The second column uses color to represent the frequency of option A, at the stable non-option B equilibrium. The third and final column, shows the standard deviation for the equilibrium reached by each subpopulation that didn’t converge on B fixation. Note that since the standard deviation is always zero, the second plots have more importance.


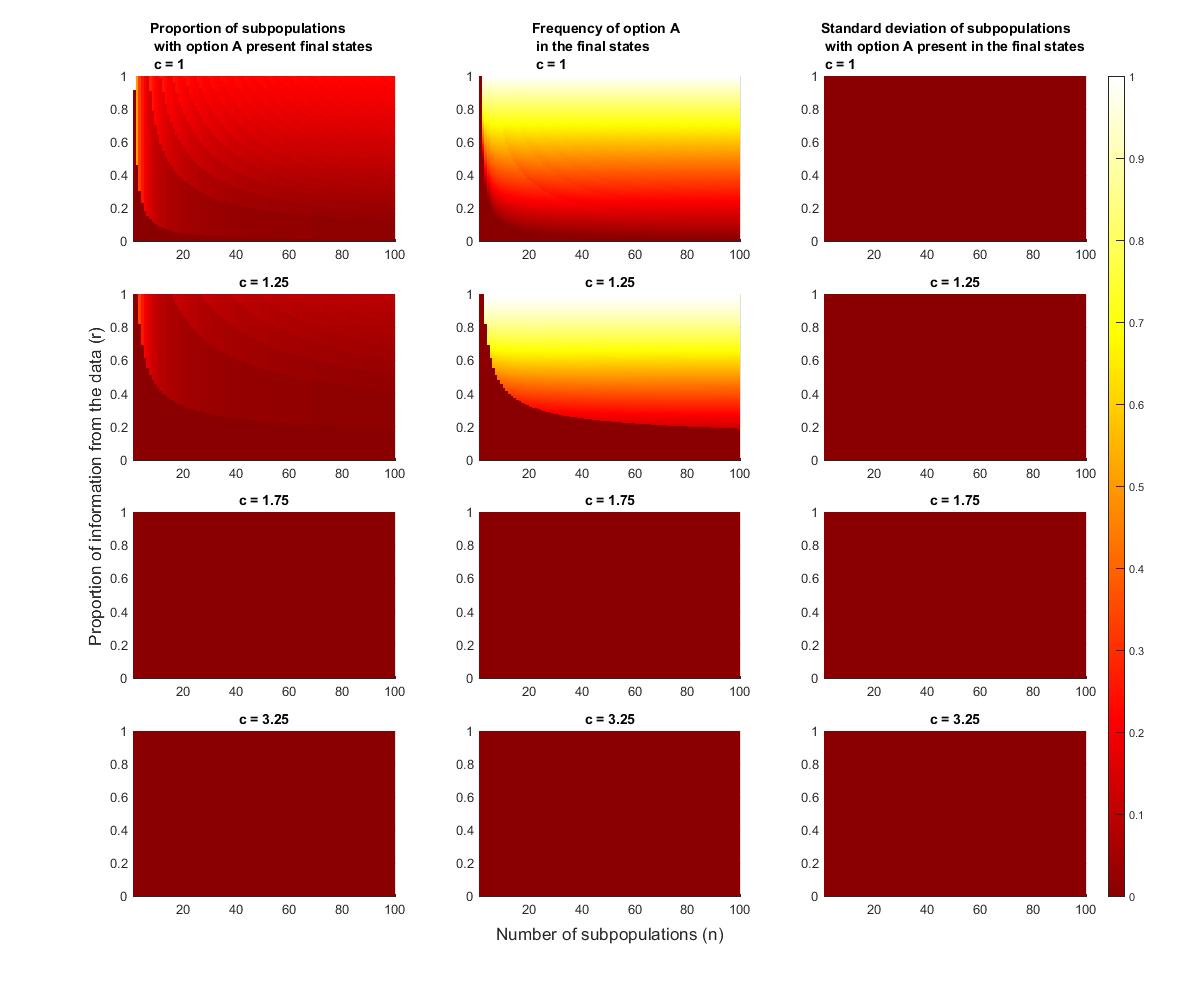


Figure 7: ($a$-$l$) Equilibrium values reached for different values of $c, r, n$. In this figure, every subpopulation is assumed to start near option B fixation, specifically $q_{i}\in(0,0.03]$ with no subpopulation starting at the exact same frequency. The first column of heatmaps, uses color to represent the proportion of subpopulation that stabilized at a non-option B fixation equilibrium. The second column uses color to represent the frequency of option A, at the stable non-option B equilibrium. The third and final column, shows the standard deviation for the equilibrium reached by each subpopulation that didn’t converge on B fixation. Note that since the standard deviation is always zero, the second plots have more importance.


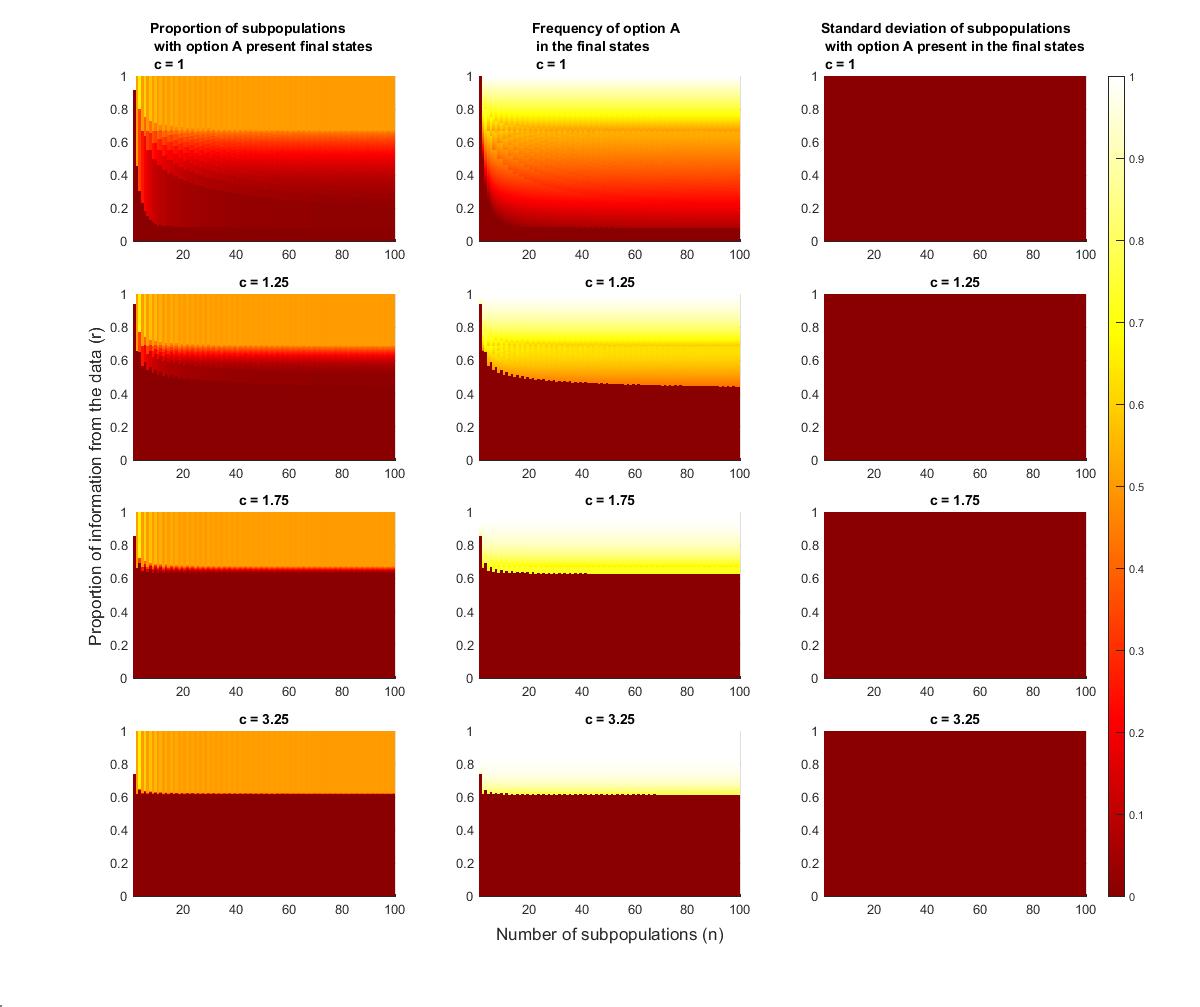


Figure 8: ($a$-$l$) Equilibrium values reached for different values of $c, r, n$. In this figure, half the subpopulations are assumed to start near option A fixation ($q_{i}\in[0.97,1))$ and the other half are assumed to start near option B fixation ($q_{i}\in(0,0.03])$. When there is an odd number of subpopulations the extra starts near option A fixation, additionally, no subpopulations start at the exact same frequency. The first column of heatmaps, uses color to represent the proportion of subpopulation that stabilized at a non-option B fixation equilibrium. The second column uses color to represent the frequency of option A, at the stable non-option B equilibrium. The third and final column, shows the standard deviation for the equilibrium reached by each subpopulation that didn’t converge on B fixation. Note that since the standard deviation is always zero, the second plots have more importance.

# Brief discussion of continuous and discrete models

The distinction between discrete and continuous traditions is briefly summarized here. Payoff-biased transmission in both models (discrete and continuous) requires different fitness landscapes to generate cultural variation, limiting its ability to explain cultural differences between geographically close populations, such as the Amish and neighboring groups. That said, norm reinforcement can stabilize discrete traditions without ecological variation, but all groups tend to converge to one of two equilibria (due to the dichotomous landscape), reducing uniqueness. Whereas norm reinforcement in a continuous context can maintain a variety of unique traditions and preserve cultural variation. In the continuous case, the final state of the system is for groups to arrange themselves symmetrically around the prior regardless of the initial state of each subpopulation (although the relative starting points of each group determines their relative end point).

Regarding the discrete model, several other projects have explored the combination of cultural transmission with biased decision-making, but their results do not fully align with our findings. For example, Boyd and Richerson (1985) introduced a direct bias that scales the observed frequency of a trait based on the direction of the bias, allowing the bias weight to vary depending on individual behavior. Their results differ from those reported here because they find that a pure-A equilibrium can arise, even in the face of a bias favoring B, which this model suggests is not possible. However, it is important to note that their implementation of biases differs from what we have presented here. In other implementations, biases exert a relative force that influences individuals' observations of others, overemphasizing traits or individuals believed to have desirable qualities. In contrast, our implementation of bias (cognitive bias) acts as phantom observations of traits that individuals believe to be optimal, representing their preconceived predisposition towards traits before making actual observations. In turn cognitive bias, in our model, functions as an absolute force that pulls individuals towards what they believe to be optimal traits at a fixed strength.

To illustrate the distinction between these biases let's consider the following scenario. Imagine flipping 10 coins that are completely unfamiliar to you, and remarkably, all 10 flips result in heads while none land on tails. When we examine the direct bias described by Boyd and Richerson (1985), it becomes clear that no matter what biases you possess, since you observed 0 tails, it is impossible to interpret the coins as fair. Consequently, you would deduce that these coins are inherently unfair and only produce heads. Now, let's explore a cognitive bias that predisposes you to believe that coins are typically fair. In this case, even though you flipped 10 consecutive heads and encountered no tails, you may still maintain the belief that the coins are fair, albeit with a diminished conviction. This is because cognitive bias doesn't incorporate into your observations multiplicatively, as direct bias does. Instead, it adds a cognitive component by generating "phantom" observations that contribute to your overall assessment.

Thus, these projects represent different kinds of bias: others implement bias that weights observations (analogous to payoff bias), while our implementation focuses on individuals' preconceived beliefs and uncertainties in their observation sample. Consequently, the findings are not competing but rather complementary since each model incorporates a different type of bias.

## Justification for tipping point as conformist transmission becomes infinitely strong

## Consider the functions $f:\left[ 0, 1 \right]\to[0, 1]$ and $g:\left[ 0, 1 \right]\to[0, 1]$ such that for any $q\in\left[ 0, 1 \right]$

## $f\left( q \right)=q'=r\frac{x\tau}{x\tau+(1-x)}$ where $r\in[0,1]$ and $\tau\in(0, \infty)$

## $g\left( q \right)=p_{A}=\frac{x^{c}}{x^{c}+\left( 1-x \right)^{c}}$ where $c\in[0, \infty)$

## Based on these functions we define the following recursive process

##

$$q_{t+1}=g\left( f\left( q_{t} \right) \right), t\in N$$

## If there exist an $q^{*}\in(0, 1)$ such that $q^{*}=g\left( f\left( x^{*} \right) \right)$ than $x^{*}$ is an equilibrium of the system:

##

$$q^{*}=\frac{f\left( q^{*} \right)^{c}}{f\left( q^{*} \right)^{c}+\left( 1-f\left( q^{*} \right) \right)^{c}}=g\left( f\left( q^{*} \right) \right)$$

##

$${\Rightarrow q}^{*}\left( f\left( q^{*} \right)^{c}+\left( 1-f\left( q^{*} \right) \right)^{c} \right)=f\left( q^{*} \right)^{c}$$

##

$$\Rightarrow f\left( q^{*} \right)^{c}\left( 1-q^{*} \right)=\left( 1-f\left( q^{*} \right) \right)^{c}q^{*}$$

##

$$\Rightarrow f\left( q^{*} \right)\left( 1-q^{*} \right)^{1/c}=\left( 1-f\left( q^{*} \right) \right)\left( q^{*} \right)^{1/c}$$

## Taking the limit as conformist tendency $(c)$ approaches infinity gives:

##

$$\lim_{c\to\infty} f\left( q^{*} \right)\left( 1-q^{*} \right)^{1/c}=\lim_{c\to\infty} \left( 1-f\left( q^{*} \right) \right)\left( q^{*} \right)^{1/c}$$

##

$$\Rightarrow f\left( q^{*} \right)\lim_{c\to\infty} \left( 1-q^{*} \right)^{1/c}=\left( 1-f\left( q^{*} \right) \right)\lim_{c\to\infty} \left( q^{*} \right)^{1/c}$$

##

$$\Rightarrow f\left( q^{*} \right)=1-f\left( q^{*} \right)$$

##

$$\Rightarrow f\left( q^{*} \right)=\frac{1}{2}$$

##

$$\Rightarrow f\left( q^{*} \right)=r\frac{{\tau q}^{*}}{{\tau q}^{*}+(1-q^{*})}=\frac{1}{2}$$

##

$${\Rightarrow q}^{*}=\frac{1}{2r\tau-\tau+1}$$

## The equilibrium, $q^{*} = \frac{1}{2r\tau-\tau+1}$ is the tipping point value (unstable equilibrium) where any initial frequency above will converge to a stable equilibrium near one. And any initial frequency below $q^{*}$will converge to zero. Notice a special case of $q^{*} = \frac{1}{2r\tau-\tau+1}$ is when $\tau=1$ (i.e., no relative payoff) in which case $q^{*} = \frac{1}{2r(1)-1+1}=\frac{1}{2r-1}$.

## Justification for equation 15

## Given that $c = 1$, the probability of adopting A ($p_{A}$) becomes equal to its perceived frequency ($q'$) and so equilibria exist where $q = q'$. There are two solutions to $q = q'$, and thus two equilibria. The first is zero since inputting zero returns zero ($q^{'}=r\cdot\frac{0\cdot\tau}{0\cdot\tau+\left( 1-0 \right)}=0$). The second requires the following algebra steps:

##

$$q^{*}=r\frac{{\tau q}^{*}}{{\tau q}^{*}+(1-q^{*})}$$

##

$${\tau q}^{*}+(1-q^{*})=r\tau$$

##

$$q^{*}\left( \tau-1 \right)=r\tau-1$$

##

$$q_{1}^{*}=\frac{r\tau-1}{\tau-1}$$

## Although $q_{1}^{*}=\frac{r\tau-1}{\tau-1}$ and $q_{2}^{*}=0$ are both equilibria, neither are necessarily stable. Conditions for stability are found through evaluating when the magnitude of the derivative for $q'$ at each equilibrium, is less than one:

##

$$f\left( q \right)=\frac{\partial}{\partial q}q^{'}=\frac{r\tau}{\left( \tau q-q+1 \right)^{2}}$$

##

$$f\left( q_{1}^{*} \right)=\frac{r\tau}{\left( \tau\left( \frac{r\tau-1}{\tau-1} \right)-\left( \frac{r\tau-1}{\tau-1} \right)+1 \right)^{2}}=\frac{1}{r\tau}$$

##

$$f\left( q_{2}^{*} \right)=\frac{r\tau}{\left( \tau\left( 0 \right)-\left( 0 \right)+1 \right)^{2}}=r\tau$$

## Notice that $\left| f\left( q_{1}^{*} \right) \right|<1$ if and only if $\frac{1}{r}<\tau$ hence $q_{1}^{*}$ is stable if $\frac{1}{r}<\tau$. Conversely $\left| f\left( q_{2}^{*} \right) \right|<1$ if and only if $\frac{1}{\tau}>r$ hence $q_{2}^{*}$ is stable if $\frac{1}{r}>\tau$. Unsurprisingly only one equilibrium is stable, but exactly which one depends on $\tau,r$. Building on the stability conditions we can find when option A persist as the majority behavior. First, we determine when $q_{1}^{*}>0.5$:

##

$$q_{1}^{*}=\frac{r\tau-1}{\tau-1}>\frac{1}{2}$$

##

$$r\tau-1>\frac{1}{2}\left( \tau-1 \right)$$

##

$$2(r\tau-1)>\left( \tau-1 \right)$$

##

$$\tau(2r-1)>1$$

##

$$\tau>\frac{1}{(2r-1)}$$

## Therefore if $\tau>\frac{1}{2r-1}$ then $q_{1}^{*}>0.5$, however, $q_{1}^{*}$ could theoretically be unstable when $\tau>\frac{1}{2r-1}$. That said, since $q_{1}^{*}$ is stable when $\tau>\frac{1}{2r}$ and $\frac{1}{2r-1}>\frac{1}{2r}$ (provided $r>0$), we conclude that when $\tau>\frac{1}{2r-1}$ then $\tau>\frac{1}{2r}$ meaning $q_{1}^{*}$ is not only stable but also greater then 0.5.
